# Copper Deficiency in Sheep with High Liver Iron Accumulation

**DOI:** 10.1155/2012/207950

**Published:** 2012-12-18

**Authors:** Isadora Karolina Freitas de Sousa, Antonio Humberto Hamad Minervino, Rejane dos Santos Sousa, Dowglish Ferreira Chaves, Herbert Sousa Soares, Isabella de Oliveira Barros, Carolina Akiko Sato Cabral de Araújo, Raimundo Alves Barrêto Júnior, Enrico Lippi Ortolani

**Affiliations:** ^1^Departamento de Clinica Médica, Faculdade de Medicina Veterinária e Zootecnia, Universidade de São Paulo, Avenida Professor Dr. Orlando Marques de Paiva 87, Cidade Universitária, 05508-270 São Paulo, SP, Brazil; ^2^Departamento de Ciências Animais, Universidade Federal Rural do Semiárido, 59.625-900 Mossoró, RN, Brazil

## Abstract

An outbreak of enzootic ataxia among sheep raised in the northeastern region of Brazil is described. Copper (Cu) deficiency was diagnosed in a herd of 56 sheep, among which five presented characteristic clinical symptoms of enzootic ataxia. The symptoms began 30 days after birth, with a clinical condition that included locomotion difficulty, limb ataxia, tremors, and continual falls. Liver biopsies were performed and blood was collected to determine hepatic and plasmatic Cu, iron (Fe), and zinc (Zn) concentration, respectively. The laboratory results showed that the animals presented low copper concentrations in the plasma and liver, without difference between the clinically healthy animals and those affected by enzootic ataxia. Even after supplementation with adequate Cu levels had been recommended, it was found on a new visit to the farm four months later that one animal still presented a clinical condition and that the hepatic Cu levels of the herd had not risen. Despite the low copper content of the diet, the high hepatic Fe levels found suggest that antagonism due to this element may have been an important factor in triggering copper deficiency in these animals, and thus, additional copper supplementation may be necessary for these animals.

## 1. Introduction

Among the minerals, Cu is an essential microelement that presents a variety of functions in animal organisms. It plays a part in the active center of more than 20 metalloenzymes, cofactors, and metalloproteins that are connected with destruction of free radicals, synthesis of connective tissues, formation of myelin and bones, pigmentation and formation of fur and wool. It also acts indirectly in hematopoiesis [1, 2].

Lack or low quantities of copper animal feed may cause a shortage of this microelement [3]. Cu deficiency is taken to be a severe nutritional problem in tropical regions, because of the low Cu concentrations in animals' diets and/or because of high concentrations of elements that are antagonistic towards Cu, such as molybdenum (Mo), sulfur (S), and iron (Fe) [4, 5].

Although it has been well established that changes to iron intake may influence Cu metabolism in animals, little importance has been attributed to Fe as a cause of Cu deficiency in ruminants under conditions of extensive rearing [6]. In northeastern Brazil, it is believed that at times of food scarcity, the greatest source of iron intake is the soil, since the animals start to graze down closer to the ground and ingest large quantities of soil, which is rich in iron [7].

The iron antagonism to Cu absorption by the intestine can occur by three mechanisms: binding of sulfide ions (S^2−^) to soluble Fe from rumen producing iron sulfide (FeS), preventing Cu to bind to S^2−^, hindering thus the absorption of this element, binding of Cu to insoluble iron compounds, and by the utilization of nonspecific transporters of multiple metals by soluble Fe, thereafter preventing the Cu to bind to this carries and be absorbed [3, 7–9].

Low Cu levels have been found in foraging animals and in the livers of ruminants in different regions of Brazil [1, 7, 10]. Enzootic ataxia is the maximum expression of Cu deficiency in lambs up to the age of 180 days and is characterized by demyelination of the central nervous system and by the symptoms of lack of coordination of the hind legs and, to a lesser extent, the forelegs, unsteadiness while walking, flaccid or spastic paralysis, and total incapacity to walk and death [3, 11]. Two types of enzootic ataxia have been described, based on the lesion site and the chronology of the condition. The congenital form is marked by destruction of the cerebral white matter and affects neonates in their first days of life. The late form is characterized by lesions of the brain stem and spinal cord motor tracts, with occurrences after the third week of life [3].

The objective of the present study was to report on the clinical and epidemiological characteristics of an outbreak of the late form of enzootic ataxia among sheep in the municipality of Mossoró, state of Rio Grande do Norte, northeastern region of Brazil.

## 2. Methodology

The outbreak occurred on a farm property located in the municipality of Mossoró, in the state of Rio Grande do Norte. The sheep were in a herd composed of 56 animals that were being raised under a semiextensive system, with grazing on native vegetation and occasionally supplementation with maize straw, algarroba, maize bran, wheat bran, and cotton seed cake. They also received common salt (NaCl), with added trace mineral supplementation (25 kg  of  salt + 1 kg of mineral trace elements). No parental administration of copper or copper-rich products on the two farm properties was carried out.

We did two visits to this farm, the first one in December, 2008 (M0) and the second one in April, 2009 (M1). Blood samples were collected by puncturing of the external jugular vein. The samples were collected in vacuum tubes (both with and without the anticoagulant EDTA) in order to obtain serum and plasma, which were stored in plastic tube and frozen at −20°C, for subsequent biochemical and mineral analysis. Whole blood samples (with EDTA) were kept under refrigeration at 4°C until a complete hemogram was produced. At the M0 34 animals were sampled of different ages and sex, and at M1 33 animals were bled.

Whole blood samples were used for determining globular volume, red and white blood counts, and hemoglobin concentration, throughout routine techniques. Serum samples were used for biochemical evaluation using an automated apparatus (Labmax 240, Labtest, Tokyo, Japan) for determination of total protein, albumin, aspartate aminotransferase, gamma-glutamyl transferase, creatinin, urea, calcium, and inorganic phosphorus, using commercial kits (Labtest, Tokyo, Japan).

During the visits at the farm, hepatic biopsies were performed by paracostal laparotomy, adapted from the technique described by Minervino et al. [12]. Approximately 1 g of fresh liver tissue was collected and immediately frozen at −20°C until analysis. All of the sick animals had hepatic samples collected at the two visits. Additionally, healthy animals at the same age were also used for comparison, two at M0 and four at the M1.

To determine mineral concentration, plasma samples were used because the serum Cu levels can be reduced due to the clotting [13]. The plasma samples were defrosted at room temperature and then homogenized and diluted using mineral free water. The liver tissue samples were initially dried at 102°C for 24 hours and then digested in an open system using nitric-perchloric acid (4 : 1), in a digester block at a maximum temperature of 200°C. Cu, Fe, Zn, and Mo concentration in plasma and liver were assayed in a plasma optical emission spectrometer with the same procedures described by Minervino et al. [14]. Hepatic mineral concentration was presented in dry matter basis, considering the dry weight of the digested samples and the dilution.

A quality assurance and control procedure were done to guarantee analytical accuracy, which involved measurement of metal concentrations in blanks (one blank every 10 samples), calculation of limits of detection (3 S.D.s of the mean blank value), and measurement of precision, throughout measurement of a standard quality control of the laboratory every 10 samples (*n* = 8). Percentage recoveries were determined primarily from this laboratory control sample. Recovery percentages were Cu (93.2% ± 6.7); Fe (92.4 ± 8.3); Zn (94.5 ± 6.2); Mo (52.0% ± 32.4). The detection limits were Cu (0.005 ppm); Fe (0.08 ppm); Zn (0.04 ppm). Due to low accuracy during the analysis, Mo determination was not considered.

## 3. Results

At the clinical attendance on the farm, the owner reported that after the first month of life, four sheep in the herd had started to present a condition of difficulty in walking, falls, and tremors. After the symptoms started, the condition worsened, with apathy, weight loss, and death. The clinical manifestation occurred only in sheep aged from 1 to 2 months.

In the physical examination on the clinically affected animals, the following symptoms were observed: difficulty in rising to a standing position; lack of coordination; frequent falls; ataxia in the hind legs and, with less severity, in the forelegs; weight loss and progressive apathy. One of the animals was permanently in a lying-down position. The animals also presented difficulty in suckling, rough fur without shine, little pigmentation, and limited body development.

Among the four animals with clinical symptoms, three of them died between seven and 20 days after the initial diagnosis. The animal that survived continued to present sequel such as difficulty in walking and poor body development.

After the initial visit, the farm owner was advised to provide supplementation for the herd using a commercial mineral mixture that contained 590 ppm of Cu per kg. At M1, four months after the first attendance, another visit was made to the farm, and it was observed that one animal presented clinical symptoms of enzootic ataxia, which were similar to the symptoms mentioned above. According to the owner, the recommended supplementation had been implemented adequately. Neither the plasma nor the hepatic mineral concentration differed between healthy and sick animals, and thus they were discussed according to the two visits.

Table 1 presents the plasmatic concentration of Cu, Fe, and Zn during the first (M0) and the second (M1) visit to the farm.  Table 2 presents the mineral concentration on the liver of animals at M0 and M1. The hematological and biochemical parameters did not vary between the diseased animals and the animals without clinical symptoms, within the same age range and were consistent with the reference values for the species.

## 4. Discussion

The clinical condition presented was similar to what has been described in the literature for late enzootic ataxia in lambs [10, 15]. Based on the information on the label of the mineral mixture that was being used on the farm, it was seen that the supplement used before the occurrences of the clinical cases contained extremely low Cu levels: 17 mg of Cu per kg of supplement, that is, around 0.0017% Cu. Tokarnia et al. [16] reported low Cu levels in the pasture land and in the livers of ruminants in a semiarid area, thus requiring adequate mineral supplementation. For animals in northeastern Brazil to have the necessary Cu intake per day, which is around 8 mg/kg of DM in the diet [1], mineral supplementation containing 0.01% of Cu is required in order to meet these animals' needs [17]. The animals in the present study were receiving Cu supplementation in quantities that were less than one tenth of the recommended levels.

The high fatality rate observed among the sheep in this study (75%) was similar to what was reported in the study by Dos Santos et al. [10], in which the incidence of enzootic ataxia among the lambs was 42.7%. In the present report, the incidence of enzootic ataxia among the lambs was 20% (4/20) and 4.5% (1/22), at the first and second visits, respectively. This indicated that the recommended supplementation reduced the incidence of clinical cases of enzootic ataxia in this herd, but did not totally prevent occurrence of this disease.

To prevent occurrences of enzootic ataxia under conditions resembling those of the present study, with high accumulation of Fe and low organic Cu values, additional Cu supplementation may be needed, since commercial mineral supplements present low Cu levels because of the risk of intoxication.

Normal Cu levels in the liver should be greater than 101 ppm, while levels of 0–50 ppm indicate deficiency [1]. In a survey conducted in the same region of Brazil, hepatic Cu levels of between 135.56 and 186.14 ppm DM were found, with a general mean of 158.45 ppm, among sheep destined for slaughter [18]. In this manner, the determinations of Cu concentration confirmed the clinical diagnosis of enzootic ataxia.

High concentrations of Zn and Mo can interfere with copper metabolism, however hepatic zinc values were below normal for the species (110–220 ppm), probably due to inefficient mineral supplementation given to the animals [3, 14, 16].

Based on the data of Suttle [3] and of Brazilian researchers [16], normal hepatic Fe levels should be maintained between 181 and 380 ppm. In a study conducted by Marques et al. [18] in the northeastern region of Brazil, the mean hepatic Fe level for sheep was 210.53 ± 121.99 ppm. Thus, high Fe accumulation in the liver was observed (1,499.9 ppm), which must have contributed towards the occurrence of the Cu deficiency because of the antagonism provoked by Fe, which diminished the availability of Cu for these ruminants. In the study by Santos et al. [10], it was found that enzootic ataxia was caused by excessive Fe intake, which may have been related to the feeding habits of sheep, which graze down close to the ground and thus may consume grass mixed with soil, thereby further raising the Fe levels.

We have no reason to believe and there was any report on the evaluable literature that another Cu antagonist, such as lead and silver, is present in this region, our results strongly suggest that the Fe is the major responsible for the Cu deficiency described. Further studies with complete mineral profile in all seasons are required to better understanding of the mineral antagonism present in the region.

In cases of zoonotic ataxia, it is not uncommon for anemia to be present [15], but we did not find any anemia among the animals of the present study. Biochemical and hematological evaluation were not useful to diagnose copper deficiency.

Plasma Cu level was considered to be lower than the reference values for the species, which range from 11 to 20 *μ*mol/L [15]. These values were also lower than the observed by Laven and Smith [13], who found a mean Cu level of 16.3 *μ*mol/L in sheep with copper deficiency. Fe plasma concentration was above of reference limits for the species (34.6–37.45 *μ*mol/L) [3]. Jones et al. [19] considered values higher than 39 *μ*mol/L excessive. Plasma Zn remains within the reference values for sheep (12.3–18.5 *μ*mol/L), besides the lower hepatic concentration of this element.

The animals in the herd of the present study all had low plasma Cu levels, and not just the ones that was clinically affected, which suggests that this herd presented a severe Cu deficiency. This corroborates the findings from the study by Tokarnia et al. [16] in the northeastern region. However, until now, there had not been any reports of occurrences of both copper deficiency and enzootic ataxia in the state of Rio Grande do Norte.

## 5. Conclusions

The clinical symptoms, observed among sheep, were compatible with what has been described in the literature for the late form of enzootic ataxia. The antagonistic action of iron contributed towards the occurrence of copper deficiency.

There were no significant biochemical or hematological abnormalities among the animals with enzootic ataxia in the present study, but the finding of organic Cu levels lower than the normal range for sheep, especially the hepatic Cu level, confirmed the diagnosis of enzootic ataxia.

In rearing systems in the semiarid zone of northeastern Brazil, additional copper supplementation may be necessary for metaphylactic treatment of enzootic ataxia, especially for extensive raised sheep were the soil and plant mineral concentration are unknown.

## Figures and Tables

**Table 1:** Mean ± standard deviation of plasma concentrations of Cu, Zn, and Fe at the two visits on the farm.

Parameter	Cu (*μ*mol/L)		Fe (*μ*mol/L)		Zn (*μ*mol/L)	
	M1	M2	M1	M2	M1	M2
Plasma	5.9 ± 1.1	5.2 ± 1.3	39.8 ± 9.9	42.5 ± 12.7	15.3 ± 3.7	19.3 ± 4.1

**Table 2:** Mean ± standard deviation of hepatic Cu, Fe, and Zn concentration on dry matter basis in sheep at the two visits on the farm.

Parameter	Cu (ppm)		Fe (ppm)		Zn (ppm)	
	M1	M2	M1	M2	M1	M2
Liver	4.1 ± 2.5	8.3 ± 2.4	1,375 ±325	1,493 ±280	69.9 ± 18.7	67.1 ± 21.2
